# Sound-localization-related activation and functional connectivity of dorsal auditory pathway in relation to demographic, cognitive, and behavioral characteristics in age-related hearing loss

**DOI:** 10.3389/fnins.2024.1353413

**Published:** 2024-03-18

**Authors:** Junzhi Wu, Shuai Nie, Chunlin Li, Xing Wang, Ye Peng, Jiaqi Shang, Linan Diao, Hongping Ding, Qian Si, Songjian Wang, Renjie Tong, Yutang Li, Liwei Sun, Juan Zhang

**Affiliations:** ^1^Department of Otorhinolaryngology Head and Neck Surgery, Beijing Chaoyang Hospital, Capital Medical University, Beijing, China; ^2^School of Biomedical Engineering, Beijing Key Laboratory of Fundamental Research on Biomechanics in Clinical Application, Capital Medical University, Beijing, China; ^3^Center of Clinical Hearing, Shandong Second Provincial General Hospital, Jinan, Shandong, China; ^4^College of Special Education, Binzhou Medical University, Yantai, Shandong, China; ^5^School of Cyber Science and Technology, Beihang University, Beijing, China; ^6^Key Laboratory of Otolaryngology, Head and Neck Surgery, Ministry of Education, Beijing Institute of Otolaryngology, Beijing, China; ^7^Department of Otolaryngology, Head and Neck Surgery, Beijing Tongren Hospital, Capital Medical University, Beijing, China

**Keywords:** aging, hearing loss, sound localization, functional magnetic resonance imaging, functional connectivity

## Abstract

**Background:**

Patients with age-related hearing loss (ARHL) often struggle with tracking and locating sound sources, but the neural signature associated with these impairments remains unclear.

**Materials and methods:**

Using a passive listening task with stimuli from five different horizontal directions in functional magnetic resonance imaging, we defined functional regions of interest (ROIs) of the auditory “where” pathway based on the data of previous literatures and young normal hearing listeners (*n* = 20). Then, we investigated associations of the demographic, cognitive, and behavioral features of sound localization with task-based activation and connectivity of the ROIs in ARHL patients (*n* = 22).

**Results:**

We found that the increased high-level region activation, such as the premotor cortex and inferior parietal lobule, was associated with increased localization accuracy and cognitive function. Moreover, increased connectivity between the left planum temporale and left superior frontal gyrus was associated with increased localization accuracy in ARHL. Increased connectivity between right primary auditory cortex and right middle temporal gyrus, right premotor cortex and left anterior cingulate cortex, and right planum temporale and left lingual gyrus in ARHL was associated with decreased localization accuracy. Among the ARHL patients, the task-dependent brain activation and connectivity of certain ROIs were associated with education, hearing loss duration, and cognitive function.

**Conclusion:**

Consistent with the sensory deprivation hypothesis, in ARHL, sound source identification, which requires advanced processing in the high-level cortex, is impaired, whereas the right–left discrimination, which relies on the primary sensory cortex, is compensated with a tendency to recruit more resources concerning cognition and attention to the auditory sensory cortex. Overall, this study expanded our understanding of the neural mechanisms contributing to sound localization deficits associated with ARHL and may serve as a potential imaging biomarker for investigating and predicting anomalous sound localization.

## 1 Introduction

Sound localization (SL) is a crucial component of the human auditory system. The SL capabilities allow human listeners to perceive their surroundings and efficiently find objects of interest, especially for objects taking place out of sight (Mungamuru and Aarabi, 2004). Problems with localization carry noteworthy ramifications. Self-reported struggles with identifying the position and motion of sound represent some of the auditory impairments that are tightly linked to the handicap experience, such as limited social interaction and diminished emotional health (Gatehouse and Noble, 2004). With declines in the peripheral auditory system and reduced central inhibition related to advancing age, the processing of auditory cues is hindered even more (Eddins and Eddins, 2018). Although spatial cues are extracted and processed to a large extent in subcortical structures, advanced processing of spatial cues requires the involvement of the auditory cortex in neural sound location encoding and integration (Zimmer and Macaluso, 2005; Razak et al., 2015; van der Heijden et al., 2018). Therefore, functional magnetic resonance imaging (fMRI), a non-invasive and widely available brain imaging method, could be applied to elucidate the age-related abnormal activation patterns of sound locations in the cortex.

Age-related hearing loss (ARHL), referred to as presbycusis, is the third leading chronic health disorder affecting the elderly aged 65 years and above after hypertension and arthritis (GBD 2015 Disease and Injury Incidence and Prevalence Collaborators, 2016). Symmetrically declining bilateral hearing sensitivity, diminished capacity for localization of sound sources, degraded ability for speech comprehension in background noise, and delayed central processing of acoustic signals are typical features of ARHL (Rakerd et al., 1998; Hazan et al., 2018). Additionally, hearing loss is more noticeable at higher acoustic frequencies, and it is expected to impact high-frequency spectral pinna cues to elevation and front/back discrimination, as well as interaural level differential cues to azimuthal position (Hopkins and Moore, 2011; Otte et al., 2013). The requirement to integrate information across impaired location cues and limited sound frequency ranges increases computational complexity, which makes SL a challenge for the auditory system, especially in hierarchical and specialized processing of cortical spatial auditory network (Zündorf et al., 2014; Cardon and Sharma, 2018; van der Heijden et al., 2019). In the current study, we aim to investigate the role of the dorsal auditory pathway in encoding SL during the passive listening phase for ARHL patients.

The human auditory system, akin to the visual system, consists of two functionally specialized pathways: a dorsal “where” pathway dealing with sound location and a ventral “what” pathway handling sound features (Alain et al., 2001; Ahveninen et al., 2013; Zündorf et al., 2016). The auditory “where” pathway traverses through the primary auditory cortex (PAC; or Heschl’s gyrus), planum temporale (PT), inferior parietal lobule (IPL), premotor cortex (PMC), and reaches prefrontal areas (Romanski et al., 1999; Arnott et al., 2004; van der Heijden et al., 2019). Past research has described the cortical auditory processing as a hierarchical series of feed-forward analysis stages from acoustic processing in the PAC to specialized processing of higher-level sound attributes (e.g., speech perception and sound location) in high-level regions (Lewald et al., 2008; Osnes et al., 2011; Battal et al., 2019). Thus, hierarchical and specialized processing of high-level areas along the auditory “where” pathway is essential in SL processing (van der Heijden et al., 2019; Sun et al., 2023).

For decades, neuroscientists have investigated the neural representations of sound locations in high-level brain regions along the auditory “where” pathway. The auditory “where” pathway’s high-level areas were initially identified by Romanski et al. (1999), who demonstrated how it projected into the monkeys’ dorsolateral prefrontal cortex (dlPFC; Brodman area 46) and frontal eye fields (FEFs; Brodman area 8). Through comparisons of SL and sound recognition, Maeder et al. (2001) reported that the IPL and posterior frontal regions were more activated by localization than by recognition. Alain et al. (2008) found analogous inferior parietal activation during an auditory spatial working memory task. Lewald et al. (2008) discovered a cortical network consisting of inferior parietal cortex, PMC, dlPFC (Brodman area 8 and 9), insula (Brodman area 48), and inferior frontal cortex (Brodman area 47) in their task-fMRI analysis of contrasts and interaction between sound locations. Recently, Sun et al. (2023) sought to decipher the neural representations of SL during passive listening in high-level areas of the human auditory “where” pathway. Their results suggested that left FEF (Brodman area 8), as a high-level region along the auditory “where” pathway, encodes sound locations by the representation of univariate opponent hemifield activation and multivariate full-field activation pattern. At present, the studies of cortical functional changes in sound localization focus on unilateral hearing loss (Vanderauwera et al., 2020; Vannson et al., 2020; Kim et al., 2021). Vannson et al. (2020) found that cortical neuroplasticity is deleterious to the functional integrity of the dorsal auditory pathway in patients with unilateral hearing loss, which further impairs the ability of SL. However, it is still unclear what role the auditory “where” pathway plays during SL in ARHL.

Since hearing loss often leads to social segregation, melancholia, anxiety, and communication issues (Livingston et al., 2017), there are evidences that it may occur 5 to 10 years before dementia (Gates et al., 2008; Lin et al., 2011; Albers et al., 2015). Although the neuropathological mechanism for the cognitive deficits in presbycusis is unclear, some studies disclosed that patients with ARHL recruit more resources concerning cognition and attention to maintain speech perception, leading to cascading cognitive effects that further affect communication, comprehension, and working memory (Xing et al., 2020). Neurocognitive assessment of the presbycusis required a comprehensive test of cognitive status, we here used the Montreal Cognitive Assessment (MoCA) to assess general cognitive function. As they require elements of selective attention, visual search, and mental flexibility, the Stroop Color-Word Test (SCWT) and Trail Making Test (TMT) are commonly used as the clinical measures of attentional control and executive functions (such as divided attention, monitoring behavior, and switching ability), which are associated with the high-level regions of the auditory “where” pathway, i.e., the parietal and frontal lobes (Zakzanis et al., 2005; Chen et al., 2018; Periáñez et al., 2021). Therefore, we conducted those neurocognitive scales to assess the cognitive function in ARHL and to identify the neuroimaging characteristics related to neurocognitive function.

Our analysis approach used both whole-brain and regions of interest (ROIs) analyses to allow comparison to previous literatures. Then, We analyzed the relationship of activation across all ROIs during SL tasks to demographics, cognitive functioning, and behavioral features. Motivated by previous findings of the compensatory mechanism caused by ARHL, we hypothesized that cognitive functions and behavioral features would be associated with the high-level regions within the auditory “where” pathway in SL during passive listening. Ultimately, with seed-to-voxel functional connectivity analysis, we investigated the links of connectivity during SL tasks to demographic, cognitive function, and behavioral features, respectively. These findings could contribute to our comprehension of the pathological mechanisms of SL in ARHL.

## 2 Materials and methods

### 2.1 Participants

Elderly patients diagnosed with ARHL were screened for inclusion in this study conducted between September 2021 and August 2023 at the Department of Otolaryngology Clinic of Beijing Chao-Yang Hospital, Capital Medical University, Beijing (China). Patients had to fulfill the following requirements to be eligible for enrollment in the study: (1) age between 60 and 75 years; (2) participants with pure tone average (PTA, calculated by averaging the pure-tone hearing thresholds at 0.5, 1.0, 2.0, and 4.0 kHz) ≥ 20 dB HL and binaural high-frequency hearing thresholds (2 kHz, 4 kHz, 8 kHz) above 20 dB HL. Twenty-two healthy participants with normal hearing (NH) from the Capital Medical University community. Inclusion criteria is participants with PTA < 20 dB HL. Exclusion criteria for patients with ARHL and the NH participants were as follows: (1) in addition to ARHL, ear diseases that affected hearing threshold and sensorineural hearing loss, including auditory neuropathy, drug-induced deafness and Meniere’s disease; (2) a history of otologic surgery or hearing aid use; (3) conductive hearing loss and asymmetric hearing loss; (4) epilepsy, structural brain damage, Parkinson’s disease, Alzheimer’s disease, major mental or neurological disorders; and (5) MRI contraindications (Xing et al., 2021).

Two NH participants and one ARHL patient were excluded from the analysis for significant excessive head movements during scanning (>3.5 mm), and one ARHL patient was excluded due to the poor quality of images. Ultimately, NH group consisted of 20 participants, and ARHL group consisted of 22 patients. All participants gave informed written consent to join this study and received monetary incentive. In this study, the main objective is to examine the linkages between the activation pattern of the auditory “where” pathway in SL tasks during passive listening and the behavioral, cognitive, and demographic traits in patients with ARHL. Therefore, the neuroimaging data of the NH group were only used for the ROIs selection of the auditory “where” pathway. This study was approved by the ethics committee at Beijing Chao-Yang Hospital, Capital Medical University.

### 2.2 Neurocognitive assessment

The SCWT, MoCA, and TMT were employed for assessing cognition through neurocognitive assessment. The MoCA appears particularly sensitive when detecting moderate cognitive impairment and evaluating overall cognitive function. Thirty items collectively make up the assessment: visuospatial/executive, naming, memory, attention, language, abstraction, delayed recall, and orientation. The SCWT tests required participants to name color of dots (SCWT-A), colors of words (e.g., word “green” printed in green color, SCWT-B) and color of printed words (e.g., word “green” printed in red color, SCWT-C) accurately and quickly. Each subtest contains 50 stimuli and should be completed as fast as possible. Both the accuracy rate and reaction time were noted. “SCWT-C_response time_ minus SCWT-B_response time_” yields the stroop interference effect time (SIE-T). “SCWT-B_response accuracy rate_ minus SCWT -C_response accuracy rate_ “was the formula to get the accuracy rate for SIE (SIE-R). SCWT is employed to probe perceptual switching ability, selective attention, and the ability to inhibit habitual response patterns (Wang et al., 2016). The TMT involves two parts. In TMT-A, participants are presented with a paper displaying 25 circled and randomly distributed Arabic numbers, and are expected to connect these numbers in ascending order. In TMT-B, subjects are expected to alternately link numbers and letters. Participants were required to draw all the lines as fast as they could without lifting their pencils, and the time taken was subsequently recorded. Information on visual search, attention, processing speed, mental flexibility, and executive function are all manifested in the TMT (Zakzanis et al., 2005).

### 2.3 Behavior test environment

**Figure 1:**
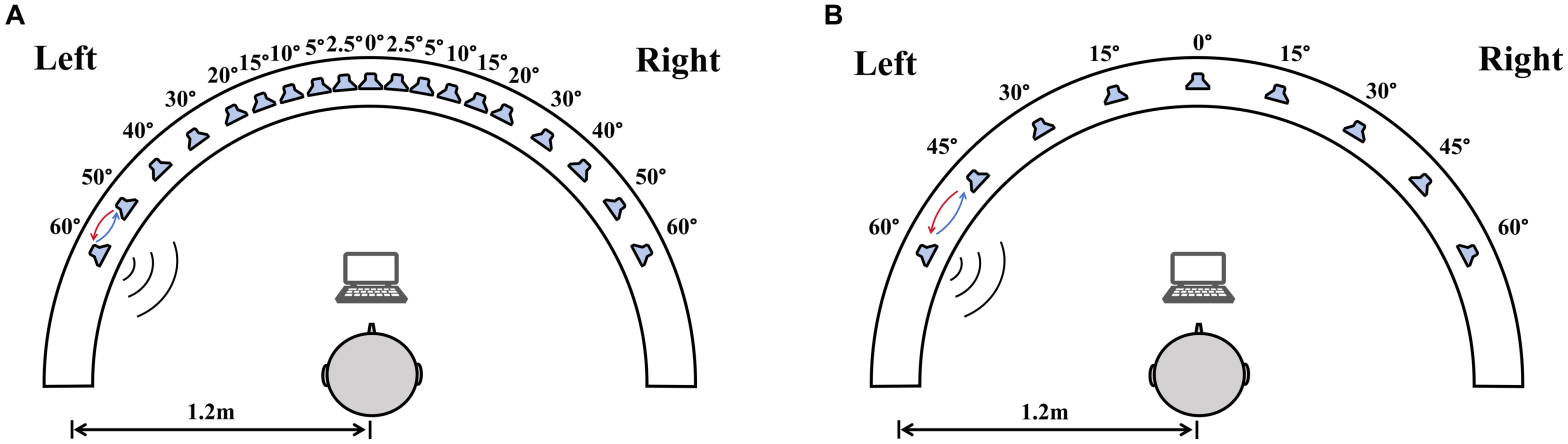
The diagram of the sound localization test. The test used a 180° horizontal arc-shaped bracket (white arcuation) with a 1.2 m radius. Participants were required to sit at the center of the bracket facing 0°. A computer monitor placed in front of the participants was used for choosing sound source. **(A)** The right–left discrimination test, nineteen loudspeakers in the bracket were placed between ±60° at the following intervals: 0°, ±2.5°, ±5°, ±10°, ±15°, ±20°, ±30°, ±40°, ±50°, and ± 60°. **(B)** The sound source identification test, nine loudspeakers were mounted on an arc spanning 120° and positioned at 15° intervals.

Sound location testing was conducted in a double-walled sound-treated booth [IAC; 2.8 m × 3.25 m with a reverberation time of 250 msec]. All loudspeakers were at ear level and a distance of 1.2 m from the center of the listener’s head. For the right–left discrimination test, nineteen loudspeakers were placed between ±60° (spanning 120°) at the following intervals: 0°, ±2.5°, ±5°, ±10°, ±15°, ±20°, ±30°, ±40°, ±50°, and ± 60° (Figure 1A). For the sound source identification test, nine loudspeakers (labeled from one to nine) that were visible to the subjects were mounted on a custom-made arc spanning 120° and positioned at 15° intervals (Figure 1B). Participants sat on a chair, facing the front loudspeaker (0°). Data was collected using a computer monitor set below the front speaker.

### 2.4 Sound localization

All stimuli used for SL testing were white noise, and the duration was 500–600 milliseconds. Stimulus levels were set to an average level of 65 dB SPL, with random roving between 59 and 71 dB SPL (i.e., ±6 dB) to minimize monaural level cues.

For the right–left discrimination test, participants participated in a 2-alternative-forced choice task. Participants used their fingers to make selections on the screen indicating the perceived side of the sound source. During blocks of ten trials, the source direction (left/right) shifted randomly, but the angular separation of the right and left speakers from the center was fixed. Each initial angle began at ±60°. If the general correctness rate reaches 70% in 10 trials, the angle decreases; conversely, the angle increases, until it achieves the minimum audible angle (MAA), meaning the least angle at which listeners can distinguish between right and left sources. MAA thresholds, which measure spatial acuity, were established as the minimum angle at which performance reached 70.9% correct. Higher MAA thresholds indicated lower ability of spatial acuity.

For the sound source identification test, subjects participated in a 9-alternative-forced choice task. Each stimulus was presented six times at each of the 9 loudspeakers for a total of 54 trials. The order of presentation of each stimulus was random. Similarly, participants used their fingers to make selections on the screen indicating the perceived side of the sound source. Localization accuracy was assessed by calculating the root-mean-square (RMS) error between the azimuth of the stimulus site and the participants’ response. Higher RMS error indicated lower ability of sound source localization accuracy.

Characteristics of SL in ARHL have high heterogeneity (deviated from the normal distribution), so the Mann–Whitney *U*-test (nonparametric test) was used to compare the behavioral features of the two groups.

### 2.5 Stimuli and procedure

**Figure 2:**
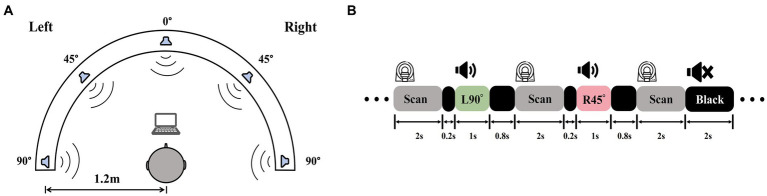
Auditory stimuli recording and experiment procedures. **(A)** A white noise clip was played from −90°, −45°, 0°, 45°, and 90° at a 1.2 m radius and recorded by a dummy head with binaural microphones. **(B)** A diagram of sparse-sampling fMRI design. The scanner paused for 2 s after each scan. During the delay, the 1-s sound clip was played following the 0.2-s blank of the previous scan, which was the 0.8-s blank ahead of the next scan. There were also blank trials with 2 s of silence.

Auditory stimuli were recorded in an isolation booth at the Department of Otorhinolaryngology, Head and Neck Surgery, Beijing Chaoyang Hospital, Capital Medical University. A dummy head wearing bilateral microphones (KU100, Georg Neumann GmbH, Germany) was placed in the center of the room. Computer-generated white noise clips were played from five different directions (−90°, −45°, 0°, 45°, 90°) at a distance of 1.2 m from the head model (Figure 2A). Five 1-s sound clips, one for each location, were recorded binaurally for the fMRI experiment. To validate the auditory stimuli, we recruited a group of five NH participants (3 females; age 22–28) and tested their ability to identify the locations of auditory stimuli presented at random orders. Three blocks of testing totaled 360 trials, with 72 trials per location. Each participant managed to attain the mean accuracy over 85%. Stimulus intensities up to 20 dB above PTA threshold, to guarantee that ARHL patients with varying degrees of hearing loss receive adequate peripheral auditory signal input.

Six task-related fMRI scans using a passive listening paradigm in this experiment. To mitigate the influence of scanner noise, a sparse-sampling fMRI structure was implemented (Figure 2B). The blood oxygenation level-dependent (BOLD) signal collecting lasted 2 s at the beginning of each trial, followed by a 2-s gap, during which a 0.2-s blank was played first, a 1-s sound clip following, and then a 0.8-s blank was ahead of the subsequent BOLD signal collection. MRI-compatible in-ear headphones (S14, Sensimetrics, United States) were employed to play the sound clips. We examined each sound clip after participants entered the scanner to make sure they could distinguish different sound positions and hear the sound clearly before the scanning began.

There were seven distinct trial types: a blank trial condition (i.e., no sound played), a catch-trial condition, and trials with five separate sound locations (−90°, −45°, 0°, 45°, and 90°). During the catch trial, the researcher would read subjects a pre-recorded random number (0 to 9). Each run had one catch trial. After the run, participants were to report the number they heard. The catch trial was incorporated to guarantee that each participant stayed alerted during the passive listening. If the participant could not provide the number, the current run’s data would be reobtained. A fixation in the center of the visual field was also requested during data collection.

Ninety trials were included in each fMRI run. A Type-1-Index-1 sequence was used to counterbalance the various trial types, which were given in a random order (Aguirre et al., 2011). After subtracting blank trials and the catch trial, there were 60 trials in total, 12 trials per site in each run. A total of 360 trials with five separate locations for sound play were conducted.

### 2.6 MRI acquisition

A Siemens 3.0 T scanner (MAGNETOM Prisma, Siemens Healthcare GmbH, Germany) with a 64-channel head coil was used for functional brain imaging. A high-resolution T1-weighted 3D MPRAGE structural scans (TR/TE = 2,300/2.27 ms, flip angle = 8°, matrix size: 192 × 256 × 256, voxel size = 1 × 1 × 1 mm^3^). Functional data were acquired through an echo-planar imaging (EPI) sequence (TR/TE = 4,000/30 ms, slice thickness = 3.5 mm, flip angle = 90°, matrix size: 64 × 64 × 35, voxel size = 3.5 × 3.5 × 3.5 mm^3^).

### 2.7 Data preprocessing

Functional preprocessing and data analysis were performed using SPM12^1^ and custom MATLAB 2017 scripts. Images were corrected for slice acquisition timing, motion, and linear trend. Motion correction was performed by estimating 6 motion parameters and regressing them out from each voxel. Then, the images were temporally smoothed using a high-pass filter with a 190 s cutoff and normalized according to the Montreal Neurological Institute (MNI) stereotaxic space. White matter (WM) and cerebrospinal fluid (CSF) signals were also eliminated from the data with WM/CSF masks and regressed from the functional data.

### 2.8 Data analyses

#### 2.8.1 Brain activation analysis

Using the general linear model, data were analyzed for every subject. For each subject, first-level statistics analysis involved generating five contrasts for each sound location against the baseline (−90°, −45°, 0°, 45°, 90°). The classic SPM hemodynamic response function was adopted to model stimulus onsets, and functional datasets were high-pass filtered with a 128 s cutoff. Then, we used a whole-brain ANOVA to estimate the effect of five different trial categories (i.e., each sound location against the baseline) on the sound localization across subjects. Specifically, regions reaching family wise error (FWE)-corrected cluster level significance at *p* < 0.05 corrected were reported.

#### 2.8.2 Regions of interest analysis

**Figure 3:**
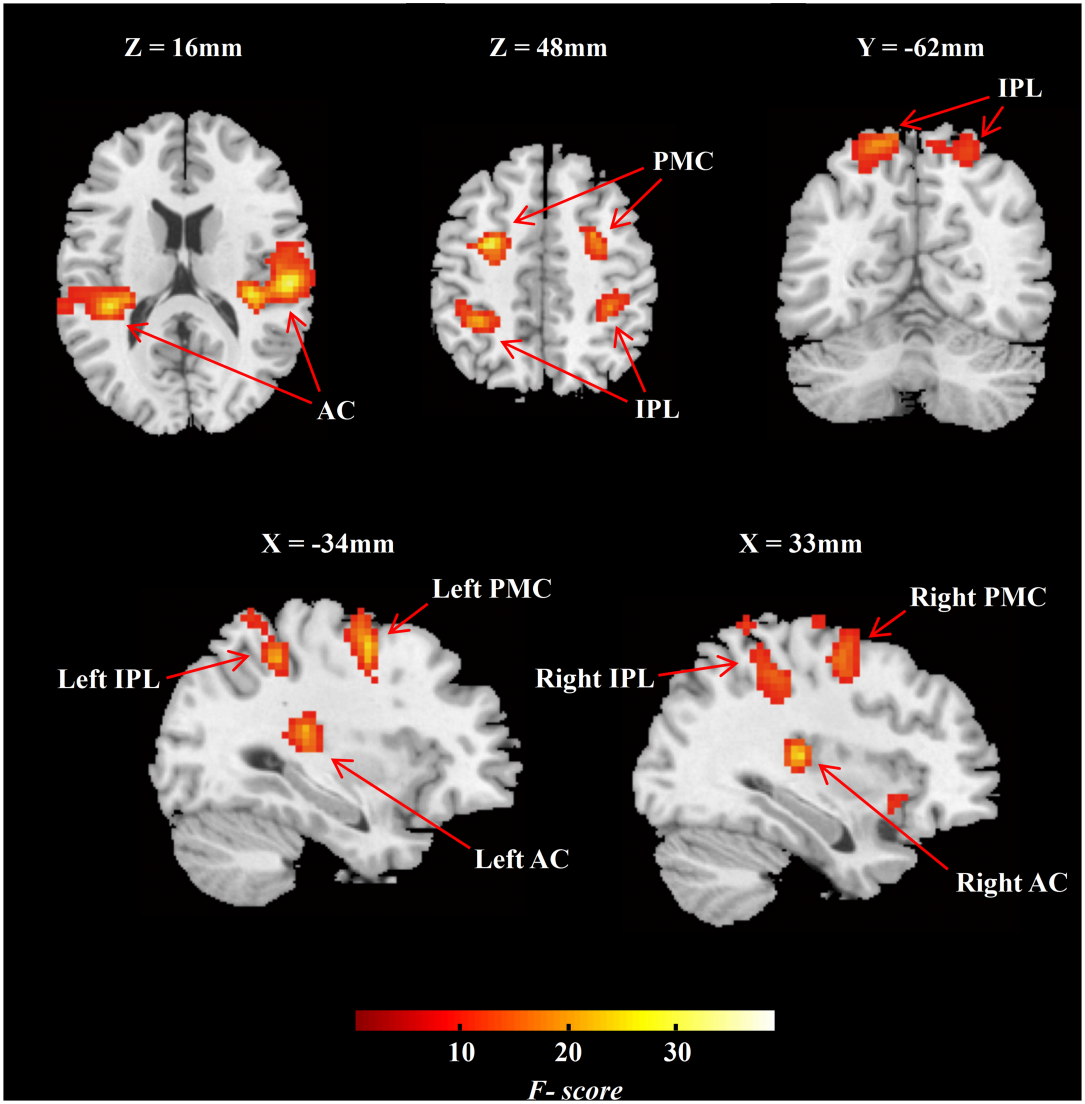
Group-level *F*-contrast map of activation across all sound locations in MNI space (NH group, *n* = 20). The map was thresholded voxel-level significance at *p* < 0.001, and a FWE-corrected cluster level significance at *p* < 0.05. AC, auditory cortex; IPL, inferior parietal lobule; PMC, premotor cortex; NH, normal hearing.

ROIs definition was based on the group-level activation map across all sound locations in NH group and earlier studies of the auditory “where” route. Specifically, we set five contrasts for each sound location against the baseline and performed the ANOVA with five trial categories for group-level activation. Activation clusters at bilateral IPL and bilateral PMC were selected due to their adjacency to previously reported auditory “where” pathway (van der Heijden et al., 2019; Figure 3). The definition of ROIs in the auditory cortex including bilateral PAC and bilateral PT were based on the Harvard–Oxford cortical structural atlas.

Then, we tested the hypothesized associations of the individual differences in ROIs response and the demographic, cognitive, and behavioral characteristics of SL in ARHL patients. Subsequently, we extracted the parameter estimates (beta weights) of each ROI in each patient with ARHL and exported them to SPSS software (version 22.0) for Spearman correlation analysis, which investigated the association between the parameter estimates and individual features. All analyses were two-tailed, with statistical significance using a Bonferroni correction to account for multiple test comparisons for each modality (adjusted threshold of *p* < 0.002 for 21 comparisons).

#### 2.8.3 Functional connectivity analysis

Connectivity analysis was analyzed using the CONN Toolbox version 18.a (Whitfield-Gabrieli and Nieto-Castanon, 2012), which is based on SPM12 routines, and was run on MATLAB 2017. Preprocessed data were submitted to CONN for seed-to-voxel analyses. Noise reduction steps of the preprocessing were below: (1) CompCor strategy used to the regression of WM and CSF regions time points with five principal components; (2) regression of time intervals at which scan-to-scan motion exceeded 1.5 mm and six rigid-body head movement parameters; (3) regression of the effects of our five sound locations blocks; and (4) temporal high-pass filtering (*f* > 0.008 Hz) to minimize signal contamination between blocks and lessen the impact of low-frequency drift.

Seed regions were consistent with ROIs of the auditory “where” pathway. In the first-level analyses, seed-to-voxel functional connectivity analyses was performed using bivariate correlations. Each ROI had its mean signal time course calculated, and the analyses were conducted by exploring correlations between the ROI signal time course and the time series of all other brain voxels. Fisher’s z-transformation of the correlation was performed to detect the voxels having notable functional connectivity to the ROIs (Han et al., 2021). In the second-level analysis, the average functional connectivity of five task conditions was assessed. Through the application of linear regression, we evaluated the relationship between task-related connectivity and behavioral, cognitive, and demographic traits in ARHL patients (voxel-level significance at *p* < 0.005, false discover rate (FDR)-corrected cluster level significance at *p* < 0.05, two-tailed).

## 3 Results

### 3.1 Demographic and clinical data

The demographic and clinical data of all participants and comparisons are shown in Table 1. Compared to the NH group, the ARHL group showed a lower ability of SL (*p* < 0.01), and significant individual differences, especially in sound source identification (RMS errors range from 2.04 to 23.13 degrees). Moreover, there was a significant decrease in scores of MoCA, SCWT-A, SCWT-B, SCWT-C, SIE-T, SIE-R, TMT-A, and TMT-B for ARHL patients.

**Table 1:** Participant demographics, cognitive and behavioral performance.

Characteristics	NH participants (*n* = 20)	ARHL patients (*n* = 22)
Age, mean ± SD (range), years	25.6 ± 2.3 (22–29)	66.72 ± 3.90 (60–74)
Female, No. (%)	14 (70.0%)	12 (54.55%)
Education, mean ± SD (range), years	17 ± 1.75 (15–20)	12.91 ± 2.58 (6–17)
PTA of left ear (range), dB HL	3.63 ± 2.55 (0–10)	39.82 ± 16.55 (18.75–70.00)
PTA of right ear (range), dB HL	4.19 ± 2.63 (0–10)	38.91 ± 15.81 (17.50–70.00)
Averaged PTA of both ears (range), dB HL	3.90 ± 2.45 (0–8.75)	39.41 ± 15.94 (20.00–70.00)
Disease duration, mean ± SD (range), years	NA	6.14 ± 6.31 (1–20)
MAA, mean ± SD (range), degree	2.50 ± 0.00 (0–2.50)	3.52 ± 1.84 (2.50–10.00)
	Z = −2.72. *p* = 0.006	
RMS error, mean ± SD (range), degree	1.63 ± 0.79 (0–3.01)	8.72 ± 5.32 (2.04–23.13)
	Z = −5.14. *p* < 0.001	
*Cognitive variables*		
MoCA, mean ± SD (range), score	29.25 ± 0.71 (28–30)	24.00 ± 4.02 (17–28)
	Z = −5.09. *p* < 0.001	
SCWT-A, mean ± SD (range), sec	20.35 ± 2.06 (18–26)	39.77 ± 19.21 (19–80)
	Z = −5.09. *p* < 0.001	
SCWT-B, mean ± SD (range), sec	21.95 ± 2.04 (18–27)	44.68 ± 16.20 (18–75)
	Z = −4.52. *p* < 0.001	
SCWT-C, mean ± SD (range), sec	35.85 ± 2.85 (31–41)	81.45 ± 33.21 (25–160)
	Z = −4.55. *p* < 0.001	
SIE-T, mean ± SD (range), sec	13.90 ± 2.34 (10–19)	37.14 ± 22.32 (7–90)
	Z = −3.70. *p* < 0.001	
SIE-R, mean ± SD (range), num	0.40 ± 0.68 (0–2)	1.95 ± 2.08 (0–7)
	Z = −2.82. *p* = 0.005	
TMT-A, mean ± SD (range), sec	20.35 ± 2.06 (19–33)	63.82 ± 34.16 (32–170)
	Z = −5.52. *p* < 0.001	
TMT-B, mean ± SD (range), sec	69.30 ± 6.91 (58–82)	233.27 ± 97.38 (98–420)
	Z = −5.54. *p* < 0.001	

Statistical significance was calculated using Mann–Whitney *U* test implemented within the SPSS22.0 software. ARHL, age-related hearing loss; MAA, minimum audible angle; RMS error, root-mean-square error; SCWT, stroop color-word test; SIE-T/R, stroop interference effects times/accuracy rate; TMT, trail making test; NA, not applicable.

### 3.2 Regions of interest

We analyzed the task-dependent group-level activation in the NH participants and in conjunction with previous studies to define ROIs for the following analyses. The NH group-level F-contrast map of activation across five angles was FWE-corrected cluster-level significance at *p* < 0.05 (Figure 3; *n* = 20). We selected two functional ROIs, including PMC and IPL (Figure 3), on the bilateral hemisphere. According to the Harvard–Oxford cortical structural atlas, the auditory cortex on bilateral hemisphere was split into PAC and PT (Figure 3). The special information on ROIs is listed in Supplementary Table S1.

### 3.3 Task-based activation was associated with demographic and cognitive variables

Spearman correlation analysis was done to examine the relationship between individual activation of ROIs and different variables (Supplementary Table S2). Task-dependent auditory “where” pathway activation was observed to correlate with cognitive function in ARHL, as a lower TMT-B score was accompanied by an increase in the activation of left PMC (*r* = −0.444, *p* = 0.038) and right PMC (*r* = −0.447, *p* = 0.038). The higher MoCA scores were positively correlated with task-dependent activation of left PMC (*r* = 0.509, *p* = 0.015), right PMC (*r* = 0.435, *p* = 0.043), and right IPL (*r* = 0.439, *p* = 0.041). Nevertheless, there were no significant associations identified between task-based activation in the auditory “where” pathway and each subsection of MoCA (Supplementary Table S2). Notably, our data demonstrates there were no significant associations identified between the severity of hearing impairment and task-based activation in the auditory “where” pathway.

### 3.4 Correlation between task-based activation and sound localization behavior

**Figure 4:**
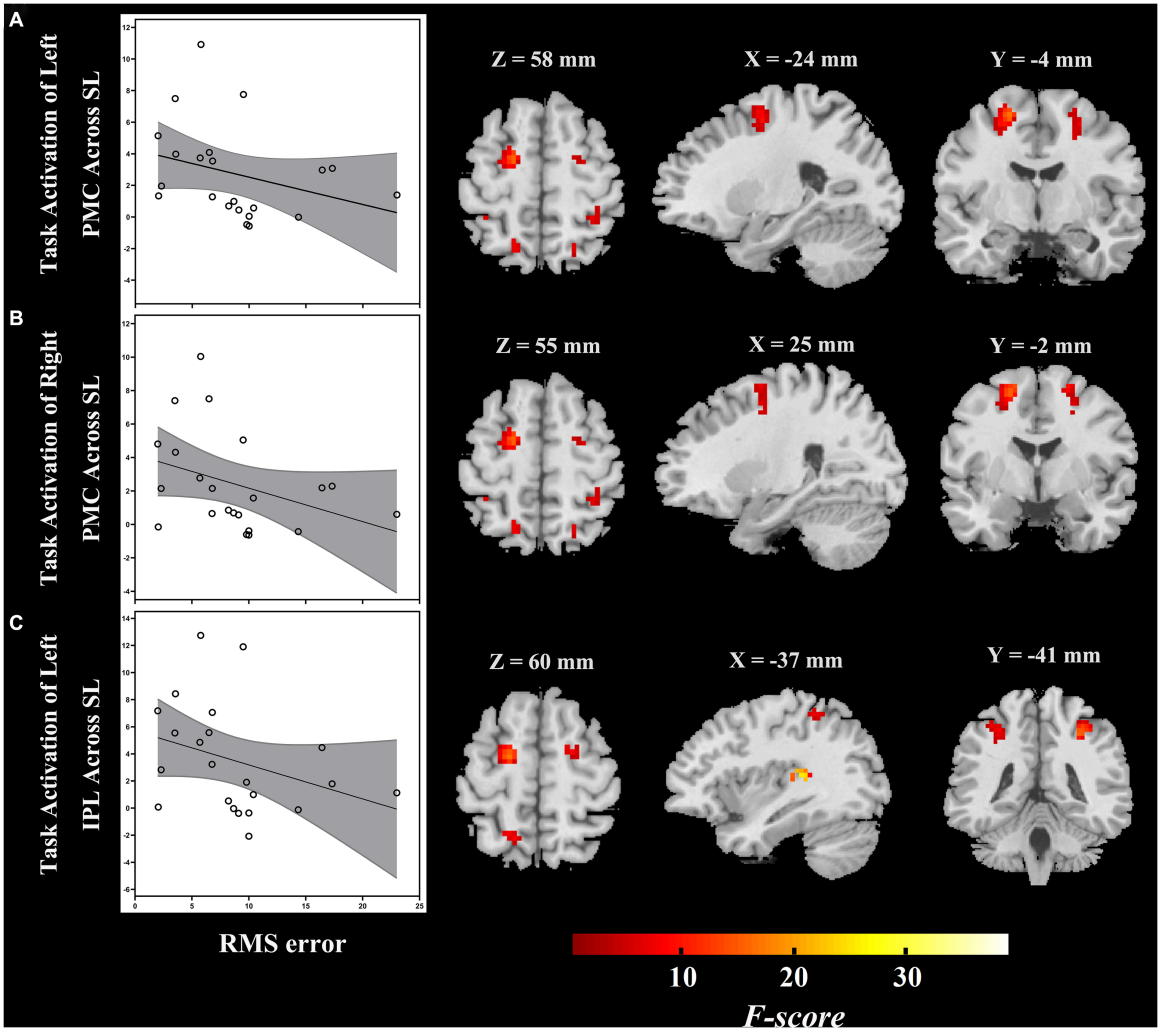
During passive listening, individuals with ARHL demonstrated reduced RMS error associated with increased task-dependent activation of **(A)** left PMC, **(B)** right PMC, and **(C)** left IPL (ARHL group, *n* = 22). Color bars indicate *F*-values, the statistical image was thresholded at *p* < 0.05 FWE-corrected. IPL, inferior parietal lobule; PMC, premotor cortex; ARHL, age-related hearing loss; RMS error, root-mean-square error; FWE-corrected, family wise error corrected.

The ROI-based correlation analysis found that the higher RMS error was associated with decreased high-level regions of the auditory “where” pathway activation in response to different sound locations, particularly bilateral PMC (left, *r* = −0.496, *p* = 0.019; right, *r* = −0.432, *p* = 0.045) and left IPL (*r* = −0.458, *p* = 0.032; though not significant after correction for multiple testing; Table 2; Figure 4). However, we detected that there was no significant association between the RMS error and the changes of task-based activity in the primary-level regions (i.e., PAC or PT) of the auditory “where” pathway. Moreover, the relationship between MAA and task-based brain activation was not associated with activation in any ROIs (Table 2).

**Table 2:** Auditory “where” pathway activation correlation to behavioral performance in patients with ARHL (ARHL group, *n* = 22).

Auditory “where” pathway	Hemisphere	Voxels	*F* value	MNI coordinates			Spearman’s correlation analysis	
				*x*	*y*	*z*	MAA	RMS error
PAC	Left	78	27.36	−45	−21	6	*r* = 0.199, *p* = 0.375	*r* = −0.135, *p* = 0.549
PAC	Right	39	30.64	45	−24	12	*r* = −0.053, *p* = 0.814	*r* = −0.155, *p* = 0.490
PT	Left	124	28.06	−39	−36	9	*r* = 0.276, *p* = 0.213	*r* = −0.091, *p* = 0.687
PT	Right	69	26.5	51	−24	12	*r* = −0.116, *p* = 0.606	*r* = −0.076, *p* = 0.736
PMC	Left	72	16.7	−18	−6	60	*r* = −0.102, *p* = 0.652	*r* = −0.496,***p* = 0.019**
PMC	Right	32	11.51	21	−3	63	*r* = 0.034, *p* = 0.881	*r* = −0.432,***p* = 0.045**
IPL	Left	59	11.37	−15	−57	60	*r* = 0.019, *p* = 0.932	*r* = −0.458,***p* = 0.032**
IPL	Right	65	16.39	30	−39	48	*r* = 0.058, *p* = 0.797	*r* = −0.334, *p* = 0.128

MNI coordinates and *F* values represent significant peak voxels of each cluster. Statistical significance was calculated using *F* tests implemented within the SPM12 software with an FWE-corrected cluster corrected *p* < 0.05. Spearman’s correlation analysis was used to analyze the correlation between average task activation of auditory “where” pathway regions and behavioral features of sound localization; *r* means the correlation coefficient; Bold *p* indicate statistical significance at *p* < 0.05. PAC, primary auditory cortex; PT, planum temporale; PMC, premotor cortex; IPL, inferior parietal lobule; MAA, minimum audible angle; RMS error, root-mean-square error; FWE-corrected, family wise error corrected; ARHL, age-related hearing loss.

### 3.5 Task-based connectivity was associated with demographic and cognitive variables

To determine whether ROIs of the auditory “where” pathway connectivity were related to demographic and cognitive variables in ARHL, a general linear regression was applied to compare functional connectivity with demographic data and cognitive measures obtained outside the scanner. We found that functional connectivity of certain ROIs and specific brain areas were significantly associated with some demographic and cognitive indices. Specifically, we found that the connectivity between right PMC and bilateral insular cortex (right, *t* = 7.10, *p* = 0.004; left, *t* = 4.11, *p* = 0.043), right IPL and right insular cortex (*t* = 8.16, *p* < 0.001), right IPL and left PAC (*t* = 5.92, *p* = 0.006), left PAC and right superior parietal lobule (SPL, *t* = 6.03, *p* = 0.003), right PAC and left SPL (*t* = 5.05, *p* < 0.001), and left PT and right SPL (*t* = 6.53, *p* = 0.003) was positively associated with education in ARHL. Our result also showed that the higher connectivity between left IPL and right PT (*t* = −4.83, *p* = 0.013) was associated with lower hearing loss duration. Furthermore, we discovered that task-dependent connectivity with the auditory “where” route did not significantly correlate with hearing impairment severity.

Examining seed-to-voxel connectivity of ROIs, we noticed that task-dependent connectivity was related to cognition in ARHL, in which the higher score of SIE-T was associated with the lower connectivity between right IPL and right angular gyrus (*t* = −4.17, *p* = 0.012), but higher connectivity between right PT and left middle frontal gyrus (MFG, *t* = 5.86, *p* = 0.046). Further, the score of TMT-B was negatively associated with the connectivity between right PMC and left primary visual cortex (*t* = −3.64, *p* = 0.043). We found that task-dependent connectivity with the auditory “where” passway did not significantly correlate with MoCA and its subscales. The details of above significant correlation are provided in Supplementary Table S3.

### 3.6 Relationship between task-based connectivity and sound localization behavior

**Figure 5:**
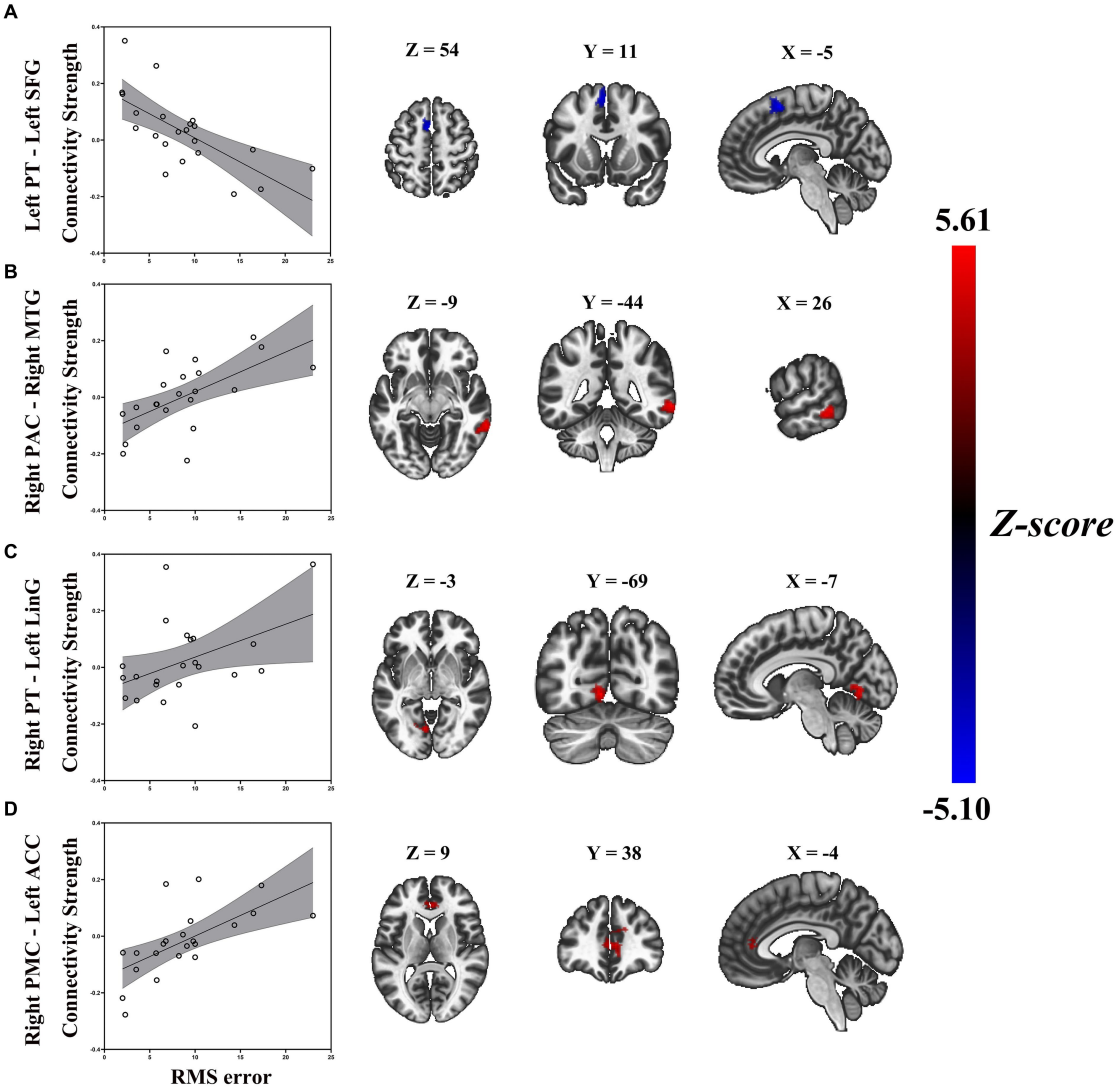
Enhanced RMS error was associated with decreased task-based connectivity of **(A)** left PT-left SFG, but increased task-based connectivity of **(B)** right PAC-right MTG, **(C)** right PT-left LiG, and **(D)** right PMC-left ACC in patients with ARHL (ARHL group, *n* = 22). Color bars indicate *t*-values, the statistical image was voxel thresholded at *p* < 0.005, FDR-corrected cluster thresholded at *p* < 0.005, two-tailed. PT, planum temporale; SFG, superior frontal gyrus; PAC, primary auditory cortex; MTG, middle temporal gyrus; LiG, lingual gyrus; PMC, premotor cortex; ACC, anterior cingulate cortex; ARHL, age-related hearing loss; RMS error, root-mean-square error; FDR-corrected, false discover rate corrected.

Similarly, a general linear regression was also applied to compare functional connectivity with SL features obtained outside the scanner. We found that connectivity between some ROIs of the auditory “where” pathway and specific brain areas availably explained variance in SL (i.e., RMS error) for ARHL patients. In patients with ARHL, four brain cluster regions whose connectivity with the ROIs of the auditory “where” pathway is related to SL during the task were revealed by the regression analysis (Table 3). Specifically, we found that connectivity between left superior frontal gyrus (SFG) and left PT (*t* = −4.46, *p* = 0.011) was negatively correlated to RMS error. In addition, connectivity between right middle temporal gyrus (MTG) and right PAC (*t* = 6.02, *p* = 0.006), left anterior cingulate cortex (ACC) and right PMC (*t* = 5.31, *p* = 0.039), and left lingual gyrus (LinG) and right PT (*t* = 5.01, *p* = 0.043) showed positive correlations with RMS error (Figure 5). Similar to task-dependent activation analysis, we found no significant correlation between MAA and functional connectivity with the ROIs after strict correction for multiple comparisons.

**Table 3:** Regression analysis for the correlations between functional brain connectivity and sound source localization accuracy in patients with ARHL (ARHL group, *n* = 22).

Seed	Brain region	Hemisphere	Cluster size (voxels)	*p*-value for cluster (FDR)	*t* value	MNI coordinates		
						*x*	*y*	*z*
Left PT	SFG	Left	251	0.011	−4.46	−6	8	56
Right PMC	ACC	Left	219	0.039	5.31	6	36	6
Right PAC	MTG	Right	250	0.006	6.02	64	−40	−10
Right PT	LinG	Left	193	0.043	5.01	−6	−68	−4

MNI coordinates and *F* values represent significant peak voxels of each cluster. Statistical significance was calculated using F tests implemented within the SPM12 software with an FWE-corrected cluster corrected *p* < 0.05. Higher RMS error indicated lower ability of sound source localization accuracy. PAC, primary auditory cortex; PT, planum temporale; PMC, premotor cortex; SFG, superior frontal gyrus; MTG, middle temporal gyrus; ACC, anterior cingulate cortex; LinG, lingual gyrus; RMS error, root-mean-square error; FDR, false discover rate; ARHL, age-related hearing loss.

## 4 Discussion

To our knowledge, we are the first to examine the neuroimaging features of the auditory “where” pathway during the state of SL task in ARHL. Our data demonstrates the abilities of SL in ARHL patients were significantly worse than that in young NH participants, with significant individual differences, especially in sound source identification. Although behavioral measurements have previously been the primary method to assess the SL ability of ARHL patients, fMRI data can provide objective information for SL and minimize the impact of man-induced factors (Rakerd et al., 1998; Otte et al., 2013). In task-dependent whole-brain analysis, we identified multiple brain regions responding to SL tasks during passive listening. Our results replicate previous findings that bilateral PAC, PT, IPL, and PMC formed the human auditory “where” pathway. Next, we defined the ROIs and examined whether individual differences in SL abilities related to the neuroimaging changes of ARHL in ROI-level analysis. Our study revealed two findings. First, individual activation in high-level regions of the auditory “where” pathway is negatively related to RMS error in ARHL, which means it’s positively related to localization accuracy. Of note, this finding should be interpreted with caution since the association did not pass correction for multiple comparison. Second, we identified strong associations between task-based connectivity and behavioral features, indicating dysfunctions of the auditory “where” pathway may serve as a potential biomarker for senescent processes of anomalous SL in ARHL.

In this research, we identified one parietal region (i.e., IPL) and one frontal region (i.e., PMC) activated in different sound locations during passive listening in NH listeners. The pathway in monkeys was initially demonstrated by Romanski et al. (1999). They discovered structural projections from PT to frontal areas after injecting chemical tracers into PT. Arnott et al. (2004) reviewed evidence from 11 neuroimaging studies on spatial hearing, which found the majority of these investigations included the appearance of the superior frontal sulcus and IPL. In our research, the PMC was detected close to the superior frontal sulcus. A recent research by Sun et al. (2023) had done a fMRI study similar to ours in NH listeners, which suggested two parietal regions, intraparietal sulcus and SPL, and one frontal region, FEF, activated in different sound locations during passive listening. Overall, brain regions of the human auditory “where” pathway found in previous studies overlap with those reported in our study.

### 4.1 Contributions of high-level regions activation to sound localization

Listeners extract binaural spatial cues from the acoustic signal relying on the proper function of their auditory periphery, thus the ARHL would be expected to degrade the localization performance of sounds (Baumgartner et al., 2016). However, this factor does not fully explain individual differences in SL of ARHL patients with similar hearing impairment. When the ARHL presents degradation of the auditory periphery, the cochlea is less effective in converting sound into neural activity, resulting in decrease of the precision of subcortical neural coding and affecting the processing of binaural spatial cues. The resultant auditory spatial signal is diminished, which may significantly affect the advanced processing of the brain cortex (McLaughlin et al., 2016). Based on both previous reports and existing data, we hypothesized that abnormal function of the auditory “where” pathway may be associated with anomalous SL in ARHL. Zatorre et al. (2002) used a speaker array permitting right–left discrimination task and sound source identification task presentation within positron emission tomography scanner. Their research revealed that the posterior superior temporal gyrus integrates binaural cues relevant to spatial position, and relays this information to parietal-lobe systems via corticocortical connections for additional processing (Zatorre et al., 2002). As compared to the right–left discrimination task, the sound source identification task required explicit spatial localization, so it needed to elicit more activity at these high-level regions (Lewis and Van Essen, 2000). In our study, we found that localization accuracy was positively associated with bilateral PMC and left IPL activation in ARHL, which indicated that high-level regions of the auditory “where” pathway may serve as a key neural biomarker underlying core feature of anomalous SL. A rest-state fMRI study by Chen et al. (2018) found that ARHL patients manifested aberrant spontaneous activity mainly in the superior temporal gyrus, precuneus, cuneus, MFG, and IPL. Our results are thus in line with previous researches (Slade et al., 2020; Xing et al., 2021, 2022), suggesting that certain functions, particularly those of the parietal and frontal cortex, may serve as an individual-specific fingerprint of ARHL and represent a characteristic indication of dysfunction in SL. As the first individualized study of SL for the “where” pathway in ARHL, the results are valuable in exploring the neuropathological mechanisms of ARHL, albeit the results of ROI analyses did not pass the *post hoc* test.

Contrary to the study hypotheses, we did not identify relationships between the activities of ROIs and MAA, even before multiple test comparisons, which might be attributed to the following three points. Firstly, this result may be attributable to the relatively small individual differences in MAA and patient numbers. Secondly, the right–left discrimination test only requires a relative judgment and can be done without computing the location itself, thus primary-level regions (i.e., PAC and PT) are sufficient to process spatial signals relevant to right–left discrimination, may not require additional processing with high-level regions (Zatorre et al., 2002). Thirdly, the sensory deprivation hypothesis postulates that ARHL patients strive to allocate more neural resources to PAC, and correspondingly, high-level regions resources decrease (Peelle and Wingfield, 2016; Slade et al., 2020; Xing et al., 2021, 2022). Therefore, for the ARHL patients in our research, the increased compensatory activation of PAC could compensate for their difficulty in discrimination ability. Additionally, the above three points same apply to interpret the association between functional connectivity of ROIs and MAA in ARHL.

### 4.2 Potential compensatory evidence: functional connectivity of the auditory “where” pathway was associated with sound source identification

Auditory deprivation can lead to cortical neuroplasticity in ARHL, whereby the “where” pathway regions deprived of input may be recruited to perform atypical functions. Studies of the sensory deprivation hypothesis in ARHL to date have typically sought to explore the possible relationship between cognitive impairment, cortical reorganization, and hearing loss (Whitson et al., 2018; Slade et al., 2020). These studies generally agree that in age-related deafness, the progressive reduction of peripheral auditory signal inputs would significantly affect the way the cerebral cortex processes these signals (Puschmann and Thiel, 2017; van der Heijden et al., 2019; Xing et al., 2021), i.e., non-auditory regions may be up-regulated to support speech perception via cortical reorganization. Therefore, we consider that this compensatory mechanism can validly explain the abnormal changes in another auditory high-level attribute (i.e., SL) to some degree during the hierarchical processing of spatial hearing signals by the auditory “where” pathway in ARHL.

For our seed area in the left PT, we found increased connectivity with the left SFG in ARHL patients with better localization accuracy. The SFG appeared in numerous spatial-hearing studies (Alain et al., 2001; Lewald et al., 2016; Zündorf et al., 2016), which was close to the high-level region (i.e., PMC) of the “where” pathway. The increased connectivity between primary-level region and high-level region of the “where” pathway indicated enhancive functional integration, which is beneficial to the fluency of SL-related resource allocation. Therefore, the connectivity between left PT to left SFG might have a main effect on sound source identification, and as a biomarker of anomalous SL.

Besides positive correlations between functional connectivity and localization accuracy, we also found the opposite pattern for some of our seed areas. Connectivity between right MTG and right PAC, left ACC and right PMC, and left LinG and right PT was negatively associated with localization accuracy. Our findings support the concept that increased connectivity of the above right seed areas in the “where” pathway are either not effective or even detrimental to sound source identification since we see a negative correlation between those connectivity and localization accuracy. This increased connectivity may arise as a compensatory mechanism in response to a loss of sound source identification. ARHL patients tend to recruit more cognitive brain resources to support auditory perception (Humes et al., 2013; Slade et al., 2020). Despite this effort to compensate for the hearing deficit, localization accuracy may severely worsen with those increased connectivity. Thus, an increase in the connectivity is likely to be either ineffective, or even detrimental to localization accuracy.

Specifically, for our seed area in right PAC, ARHL patients with poorer localization accuracy showed enhanced connections with right MTG. It is well known that MTG plays a key role in the discrimination of phonemes and semantic perception (Rogalsky et al., 2022). If these increased cortical control were an effective compensatory response, we would expect greater functional integration and fluency in the auditory cortex, which is conducive to auditory perception in the ARHL but may not be sufficient to facilitate higher-order processing for sound source identification. With the seed set at right PT, we found that increased connectivity with left LinG was associated with worsening localization accuracy in ARHL. It is well acknowledged that the LinG performs a significant part in the visual regulation system based on prior research (Kitada et al., 2010; Harpaintner et al., 2020). Abnormalities in the LinG are thought to be related to the impairment of selective attention in visual tasks (Desseilles et al., 2009). Xing et al. (2021) firstly reported aberrant functional connectivity related to LinG in ARHL patients, and indicated that with the gradual increase in the degree of hearing loss, there were permanent changes in the cross-mode functional connectivity between visual and auditory sensory areas might exist in ARHL. Combing with the above studies, our finding of increasing connectivity between left LinG and right PT might be interpreted that the poorer sound source identification could lead to the compensatory increase of audiovisual connectivity, which supported the somatosensory cross-modal reorganization in ARHL. When taking the right PMC as the seed area, we observed poorer sound source identification in ARHL associated with enhanced connections with left ACC. The ACC forms part of the cingular-opercular network, which has a broad role in learning, cognition and auditory attention (Geranmayeh et al., 2017). Fitzhugh et al. (2019) had done a resting-state fMRI in ARHL, which showed the level of hearing loss was positively correlated with connectivity from right PAC to the ACC, it might serve as a compensatory mechanism for hearing impairment. Our findings demonstrate that the compensatory increase of connectivity between left ACC and right PMC is in response to degraded processing of auditory spatial cues in ARHL with poorer sound source identification when additional attentional resources are required to maximize sound source identification performance.

### 4.3 Individual-specific regions activation and brain connectivity of the auditory “where” pathway was associated with demographic and cognitive variables

Our findings provide neuroimaging evidence for the sensory deprivation hypothesis and support the idea of cortical alterations in ARHL. Previous research suggested that the deteriorated performance in executive function measurements frequently occurred in early cognitive decline, which is associated with the frontal–parietal executive network (Caravaglios et al., 2015). Currently, TMT-B has proven to be sensitive in detecting cognitive function impairment, and its results in ARHL patients prompt the existence of cognitive deficits (Zakzanis et al., 2005). We demonstrated that the activation of the bilateral PMC was negatively correlated with TMT-B scores, favoring the association between PMC and cognitive function (Kragel et al., 2018).

As a high-level brain region significantly correlated with decreased sound identification, the PMC also contribute to executive function. Evidence indicates that the increased recruitment of compensatory cognitive resources in frontal regions during passive listening was generalized recruitment as opposed to specific motor compensation (Du et al., 2016; Slade et al., 2020). Our study found that increased connectivity between the right PMC and left primary visual cortex was associated with decreased TMT-B scores, indicating the crossing of multiple sensory and motor domains in cognitive compensation and neural upregulation. The structure of the angular gyrus was also previously associated with changes in energy metabolism, which predicted a decline in overall cognitive function (Jagust et al., 2006). In this study, we observed a significantly positive relationship between the performance on SIE-T and the task-dependent connectivity between the right IPL and right angular gyrus in ARHL. One potential interpretation for this relationship is the binding role of the angular gyrus between different domains and processing streams that are involved in multiple cognitive functions. Enhanced connectivity of these regions may fertilize this binding function and thus increase the interference effects of the SCWT. Furthermore, the enhancement of task-dependent connectivity between right PT and left MFG was associated with the worse performance on SIE-T in our sample of ARHL patients. It is not clear whether this could contribute to the reallocation of effortful attention resources, and/or an important decompensatory sign existing in ARHL patients with cognition dysfunction. More studies utilizing cognition and neuroimaging need further exploration in the future.

Socioeconomic studies have suggested that educational background is strongly associated with ARHL, where those with lower education have a higher risk of hearing loss (Tsimpida et al., 2019). Healthy elderly with higher years of education were related to greater gray matter volume in the superior temporal gyrus, IC and ACC, and were less susceptible to age-related cognitive changes (Arenaza-Urquijo et al., 2013). Bivariate correlations between ROIs in the auditory “where” pathway and education were investigated by seed-to-voxel functional connectivity analysis, in which we found that a significant proportion of ROIs (5/8) showed positive correlations between connectivity and education, suggesting that education facilitates functional integration in cortical auditory “where” pathway. In addition, a rest-state fMRI study for healthy elderly reporting education was positively related to the functional connectivity between the ACC and the frontal regions as well as parietal and temporal regions, indicating enhancement of connectivity of those regions appears as one of the mechanisms underlying education-related reserve (Arenaza-Urquijo et al., 2013). These results reinforce the perspective that more years of schooling trigger a smoother processing of neural interaction (Alvares Pereira et al., 2022). Although numerous research on neural plasticity resulting from hearing impairment have been conducted, the influence of compromised audition on the auditory cortex and the potential impact of hearing loss durations on the cerebral cortex of elderly people are still not fully understood. We then found that the reduction of task-dependent connectivity between left IPL and right PT was correlated with the length of hearing loss, but has no significant relevance to its severity. Reduced auditory input impairs sensory integrity and clearly attenuates object-recognition abilities. In the task-related fMRI scans, we gave sound stimuli at an intensity of 30 dB above the mean auditory threshold, which provided adequate peripheral signal stimulation and thus might facilitate the smoothing out of the relationship between the degree of hearing loss and the neural activity of the central cortex in ARHL patients. During the input of reduced peripheral acoustic signal, auditory and nonauditory cognitive resources will be progressively depleted over time, which in turn leads to a reorganization of the auditory cortex (Fitzhugh et al., 2019; Alvares Pereira et al., 2022), decreasing the connection between primary and high-level regions. Therefore, in this study, the duration of hearing loss may be able to better explain the neuroplasticity of the cortical auditory “where” pathway in ARHL patients than the degree of hearing loss. Our data demonstrates no correlation of task-based ROIs activation with demographic data, but task-based connectivity of some ROIs showed correlations with education and disease duration in patients with ARHL. This may be due to the efficiency of brain-region interactions involving the auditory “where” pathway is more susceptible to demographic variables (e.g., education and disease duration), relative to the degree of activation in the sound localization tasks of passive listening for patients with ARHL.

### 4.4 Limitation and future study

A primary limitation of this fMRI study is our relatively small sample size considering the known heterogeneity of ARHL patients. Although we identified activations in regions consistent with previous neuroimaging studies of the auditory “where” pathway, our ability to identify the relationship of ROIs activation in a state of SL tasks to cognitive functioning and localization accuracy was relatively limited. Of note, the associations with functional connectivity we identify are robust, adding validity to our ability to identify key neural biomarkers underlying core features of anomalous SL. Second, we only used a passive listening paradigm, without recording behavioral measures during the scanning. Therefore, future research could add active listening paradigm and record participants’ behavioral measures during the scanning, which might give us more information on the pathomechanism of SL in ARHL.

## 5 Conclusion

This study provides new findings revealing that individualized localization accuracy is positively associated with activities in high-level regions of the auditory “where” pathway, suggesting the reduction of specialized processing with high-level regions could preferentially affect the sound source identification in ARHL. Our study also gives some effective evidences for the compensatory mechanism based on the sensory deprivation hypothesis in ARHL. We found the increased connectivity between left PT to left SFG was associated with better localization accuracy, indicating the enhanced fluency of primary-level region to high-level region in the auditory “where” pathway is beneficial to auditory spatial processing, and might have a favorable effect on sound source identification. However, increased connectivity between right MTG and right PAC, left ACC and right PMC, and left LinG and right PT may be either not effective or detrimental to sound source identification, which may be a crucial decompensatory sign in ARHL. Brain activation and connectivity of certain ROIs in SL were associated with education, hearing loss duration, and cognitive function, which were helpful for further understanding the compensatory cortical reorganization and mechanism of neural plasticity in ARHL. Our comprehension of the cortical processes behind SL deficiencies linked to ARHL has been expanded by this research, which may also serve as a potential imaging biomarker to study and forecast anomalous SL in the future.

## Data availability statement

The original contributions presented in the study are included in the article/Supplementary material, further inquiries can be directed to the corresponding authors.

## Ethics statement

The studies involving humans were approved by the Ethical Committee of Beijing Chao-Yang Hospital, Capital Medical University (no. 2021-4-23-2). The studies were conducted in accordance with the local legislation and institutional requirements. The participants provided their written informed consent to participate in this study. Written informed consent was obtained from the individual(s) for the publication of any potentially identifiable images or data included in this article.

## Author contributions

JW: Data curation, Formal analysis, Investigation, Methodology, Writing – original draft. SN: Data curation, Investigation, Methodology, Writing – original draft. CL: Data curation, Formal analysis, Investigation, Methodology, Project administration, Supervision, Writing – original draft, Writing – review & editing. XW: Data curation, Formal analysis, Methodology, Writing – review & editing. YP: Writing – original draft. JS: Investigation, Writing – review & editing. LD: Investigation, Methodology, Writing – review & editing. HD: Investigation, Writing – review & editing. QS: Writing – review & editing. SW: Writing – review & editing. RT: Data curation, Writing – review & editing. YL: Writing – review & editing. LS: Data curation, Formal analysis, Investigation, Methodology, Project administration, Supervision, Writing – original draft. JZ: Data curation, Investigation, Methodology, Project administration, Supervision, Writing – review & editing.

## Footnotes

^1^ http://www.fil.ion.ucl.ac.uk/spm
