# Supplementary material for: Sound-localization-related activation and functional connectivity of dorsal auditory pathway in relation to demographic, cognitive, and behavioral characteristics in age-related hearing loss
Source: Front Neurosci. 2024 Mar 18;18:1353413. doi: 10.3389/fnins.2024.1353413 (PMC10982313; doi:10.3389/fnins.2024.1353413)
Supplement: Supplementary file 1 [file Table_1.doc]

**Supplementary Table S1 Coordinates of Regions of Interest in MNI 2 mm space in NH subjects (NH group, n = 20).**

| ROI | Size range* (voxels) | Maximum F score** | X (mm) | Y (mm) | Z (mm) |
| --- | --- | --- | --- | --- | --- |
| Left PAC | 309 | 19.67 | －45 | －25 | 9 |
| Right PAC | 282 | 31.05 | 36 | －27 | 12 |
| Left PT | 565 | 26.68 | －42 | －33 | 14 |
| Right PT | 440 | 24.61 | 39 | －30 | 10 |
| Left IPL | 243 | 22.37 | －36 | －42 | 51 |
| Right IPL | 229 | 19.25 | 36 | －36 | 48 |
| Left PMC | 238 | 39.72 | －27 | －3 | 54 |
| Right PMC | 240 | 26.94 | 24 | 1 | 52 |

* PAC and PT were defined structurally with fixed size by the Harvard-Oxford cortical atlas. Other ROIs were defined functionally for group-level activation of participants with normal hearing

** In order to show the approximate position of each ROI, the maximum F score and its coordinates were calculated from the average activation F map at group level (n = 20).

**Abbreviations:** PAC, primary auditory cortex; PT, planum temporale; PMC, premotor cortex; IPL, inferior parietal lobule; NH, normal hearing.
