# Supplementary material for: Sound-localization-related activation and functional connectivity of dorsal auditory pathway in relation to demographic, cognitive, and behavioral characteristics in age-related hearing loss
Source: Front Neurosci. 2024 Mar 18;18:1353413. doi: 10.3389/fnins.2024.1353413 (PMC10982313; doi:10.3389/fnins.2024.1353413)
Supplement: Supplementary file 2 [file Table_2.doc]

**Supplementary Table S2.** Auditory “where” pathway activation correlation to demographics and cognitive characteristics in patients with ARHL (ARHL group, n = 22).

| Auditory “where” pathway | Spearman's correlation analysis | | | | | | | | | | | | | | | | | | |
| --- | --- | --- | --- | --- | --- | --- | --- | --- | --- | --- | --- | --- | --- | --- | --- | --- | --- | --- | --- |
|  | Education | PTA of better ear | PTA of worse ear | Disease duration | MoCA-total | MoCA-visuospatial/executive | MoCA-naming | MoCA-attention | MoCA-language | MoCA-abstraction | MoCA-delayed recall | MoCA-orientation | SCWT-A | SCWT-B | SCWT-C | SIE-T | SIE-R | TMT-A | TMT-B |
| Left-PAC | *r* = 0.089, *p* = 0.694 | *r* = 0.024, *p* = 0.916 | *r* = 0.199, *p =* 0.375 | *r* = 0.253, *p =* 0.257 | *r* = 0.179, *p =* 0.425 | *r* = 0.013, *p* = 0.954 | *r* = 0.145, *p* = 0.520 | *r* = 0.030, *p =* 0.894 | *r* = 0.124, *p =* 0.582 | *r* = 0.204, *p =* 0.362 | *r* = 0.353, *p =* 0.107 | *r* = -0.005, *p =* 0.375 | *r* = 0.269, *p =* 0.226 | *r* = 0.356, *p =* 0.104 | *r* = 0.126, *p =* 0.577 | *r* = -0.080, *p =* 0.723 | *r* = -0.300, *p =* 0.175 | *r* = -0.233, *p =* 0.297 | *r* = -0.125, *p =* 0.580 |
| Right-PAC | *r* = -0.029, *p* = 0.897 | *r* = 0.052, *p* = 0.820 | *r* = 0.230, *p =* 0.302 | *r* = 0.088, *p =* 0.697 | *r* = 0.269, *p =* 0.227 | *r* = 0.094, *p* = 0.679 | *r* = 0.110, *p* = 0.627 | *r* = 0.081, *p =* 0.719 | *r* = 0.095, *p =* 0.674 | *r* = 0.260, *p =* 0.242 | *r* = 0.339, *p =* 0.123 | *r* = 0.257, *p =* 0.247 | *r* = 0.132, *p =* 0.558 | *r* = 0.202, *p =* 0.368 | *r* = 0.037, *p =* 0.871 | *r* = -0.023, *p =* 0.918 | *r* = -0.210, *p =* 0.347 | *r* = -0.114, *p =* 0.615 | *r* = -0.099, *p =* 0.662 |
| Left-PT | *r* = 0.124, *p* = 0.582 | *r* = 0.016, *p* = 0.944 | *r* = 0.194, *p =* 0.388 | *r* = 0.226, *p =* 0.311 | *r* = 0.089, *p =* 0.693 | *r* = -0.027, *p* = 0.906 | *r* = 0.160, *p* = 0.477 | *r* = -0.044, *p =* 0.388 | *r* = 0.087, *p =* 0.701 | *r* = 0.186, *p =* 0.408 | *r* = 0.209, *p =* 0.351 | *r* = 0.014, *p =* 0.949 | *r* = 0.338, *p =* 0.124 | *r* = 0.383, *p =* 0.078 | *r* = 0.164, *p =* 0.465 | *r* = -0.037, *p =* 0.869 | *r* = -0.321, *p =* 0.145 | *r* = -0.240, *p =* 0.281 | *r* = -0.146, *p =* 0.516 |
| Right-PT | *r* = -0.105, *p* = 0.643 | *r* = 0.101, *p* = 0.654 | *r* = 0.274, *p =* 0.216 | *r* = 0.189, *p =* 0.399 | *r* = 0.155, *p =* 0.492 | *r* = 0.064, *p* = 0.776 | *r* = 0.075, *p* = 0.740 | *r* = 0.014, *p =* 0.949 | *r* = 0.012, *p =* 0.959 | *r* = 0.316, *p =* 0.152 | *r* = 0.216, *p =* 0.334 | *r* = 0.267, *p =* 0.230 | *r* = 0.157, *p =* 0.486 | *r* = 0.246, *p =* 0.270 | *r* = 0.123, *p =* 0.587 | *r* = 0.062, *p =* 0.783 | *r* = -0.145, *p =* 0.521 | *r* = -0.057, *p =* 0.801 | *r* = -0.018, *p =* 0.938 |
| Left-PMC | *r* = 0.246, *p* = 0.269 | *r* = -0.231, *p* = 0.301 | *r* = -0.082, *p =* 0.717 | *r* = -0.038, *p =* 0.866 | *r* = 0.509, p = 0.015 | *r* = 0.313, *p* = 0.155 | *r* = 0.275, *p* = 0.215 | *r* = 0.366, *p =* 0.094 | *r* = -0.278, *p =* 0.210 | *r* = 0.223, *p =* 0.319 | *r* = 0.307, *p =* 0.165 | *r* = 0.266, *p =* 0.231 | *r* = -0.084, *p =* 0.709 | *r* = -0.009, *p =* 0.968 | *r* = -0.147, *p =* 0.513 | *r* = -0.157, *p =* 0.486 | *r* = -0.208, *p =* 0.353 | *r* = -0.104, *p =* 0.645 | *r* = -0.444, p = 0.038 |
| Right-PMC | *r* = 0.282, *p* = 0.204 | *r* = -0.211, *p* = 0.346 | *r* = -0.275, *p =* 0.215 | *r* = 0.011, *p =* 0.962 | *r* = 0.435, p = 0.043 | *r* = 0.345, *p* = 0.116 | *r* = 0.251, *p* = 0.260 | *r* = 0.267, *p =* 0.023 | *r* = 0.149, *p =* 0.509 | *r* = -0.222, *p =* 0.319 | *r* = 0.276, *p =* 0.214 | *r* = 0.211, *p =* 0.345 | *r* = -0.005, *p =* 0.984 | *r* = 0.086, *p =* 0.702 | *r* = -0.116, *p =* 0.608 | *r* = -0.174, *p =* 0.438 | *r* = -0.277, *p =* 0.211 | *r* = -0.184 , *p =* 0.414 | *r* = -0.447, ***p =* 0.037** |
| Left-IPL | *r* = 0.230, *p* = 0.303 | *r* = -0.275, *p* = 0.215 | *r* = -0.091, *p =* 0.687 | *r* = -0.110, *p =* 0.625 | *r* = 0.401, *p =* 0.064 | *r* = 0.178, *p* = 0.429 | *r* = 0.265, *p* = 0.234 | *r* = 0.266, *p =* 0.023 | *r* = 0.284, *p =* 0.201 | *r* = -0.219, *p =* 0.317 | *r* = 0.214, *p =* 0.340 | *r* = 0.309, *p =* 0.162 | *r* = 0.045, *p =* 0.841 | *r* = 0.037, *p =* 0.869 | *r* = -0.063, *p =* 0.782 | *r* = -0.083, *p =* 0.713 | *r* = -0.347, *p =* 0.114 | *r* = -0.182 , *p =* 0.419 | *r* = -0.406, *p =* 0.061 |
| Right-IPL | *r* = 0.150, *p* = 0.506 | *r* = -0.101, *p* = 0.654 | *r* = 0.052, *p =* 0.818 | *r* = 0.074, *p =* 0.743 | *r* = 0.439, p = 0.041 | *r* = 0.246, *p* = 0.271 | *r* = 0.384, *p* = 0.078 | *r* = 0.247, *p =* 0.268 | *r* = 0.223, *p =* 0.318 | *r* = 0.292, *p =* 0.179 | *r* = 0.222, *p =* 0.321 | *r* = 0.345, *p =* 0.110 | *r* = 0.130, *p =* 0.563 | *r* = 0.196, *p =* 0.382 | *r* = 0.047, *p =* 0.836 | *r* = -0.030, *p =* 0.895 | *r* = -0.260, *p =* 0.243 | *r* = -0.089 , *p =* 0.694 | *r* = -0.347, *p =* 0.113 |

**Note:** Statistical significance was calculated using F tests implemented within the SPM12 software with an FWE-corrected cluster corrected *p* < 0.05. Spearman's correlation analysis was used to analyze the correlation between average task activation of auditory “where” pathway regions and behavioral features of sound localization; *r* means the correlation coefficient; Bold P indicates statistical significance at *p* < 0.05; adjusted threshold of p <0.002 for 21 comparisons.

**Abbreviations:** PAC, primary auditory cortex; PT, planum temporale; PMC, premotor cortex; IPL, inferior parietal lobule; MAA, minimum audible angle; RMS error, root-mean-square error; FWE-corrected: family wise error corrected; ARHL, age-related hearing loss.
