# Supplementary material for: Sound-localization-related activation and functional connectivity of dorsal auditory pathway in relation to demographic, cognitive, and behavioral characteristics in age-related hearing loss
Source: Front Neurosci. 2024 Mar 18;18:1353413. doi: 10.3389/fnins.2024.1353413 (PMC10982313; doi:10.3389/fnins.2024.1353413)
Supplement: Supplementary file 3 [file Table_3.doc]

**Supplementary Table S3.** Regression analysis for the correlations of functional brain connectivity to demographic and cognitive features in patients with ARHL (ARHL group, n = 22).

| Characteristics | Seed | Brain region | Hemisphere | Cluster size (voxels) | P-value for cluster (FDR) | t value | MNI Coordinates | | |
| --- | --- | --- | --- | --- | --- | --- | --- | --- | --- |
|  |  |  |  |  |  |  | *x* | *y* | *z* |
| Education | Right-PMC | IC | Right | 327 | 0.004 | 7.10 | 32 | 16 | －18 |
|  | Right-PMC | IC | Left | 185 | 0.043 | 4.11 | －42 | 18 | 6 |
|  | Right-IPL | IC | Right | 540 | <0.001 | 8.16 | 36 | 14 | －18 |
|  | Right-IPL | PAC | Left | 258 | 0.006 | 5.92 | －44 | －20 | 8 |
|  | Left-PAC | SPL | Right | 283 | 0.003 | 6.03 | 18 | －60 | 62 |
|  | Right-PAC | SPL | Left | 330 | <0.001 | 5.05 | －24 | －80 | 30 |
|  | Left-PT | SPL | Right | 278 | 0.003 | 6.53 | 18 | －60 | 64 |
| Hearing loss duration | Left-IPL | PT | Right | 263 | 0.013 | －4.83 | 58 | －16 | 8 |
| SIE-T | Right-IPL | AG | Right | 264 | 0.012 | －4.17 | 36 | －72 | －16 |
|  | Right-PT | MFG | Left | 154 | 0.046 | 5.86 | －30 | 10 | 50 |
| TMT-B | Right-PMC | V1 | Left | 236 | 0.043 | －3.64 | －8 | －74 | 12 |

**Note:** MNI coordinates and F values represent significant peak voxels of each cluster. Statistical significance was calculated using F tests implemented within the SPM12 software with an FWE-corrected cluster corrected *P* < 0.05. Higher RMS error indicated lower ability of sound source localization accuracy.

**Abbreviations:** SIE-T, Stroop interference effect time; TMT-B, Trail Making Test part B; PAC, primary auditory cortex; PT, planum temporale; IPL, inferior parietal lobule; PMC, premotor cortex; IC, insular cortex; MFG, middle frontal gyrus; SPL, superior parietal lobule; AG, angular gyrus; V1, Primary visual cortex; FDR, false discover rate; ARHL, age-related hearing loss.
